# Revealing inconsistencies between Epworth scores and apnea-hypopnea index when evaluating obstructive sleep apnea severity: a clinical retrospective chart review

**DOI:** 10.3389/fneur.2024.1387924

**Published:** 2024-06-10

**Authors:** Dylan Amiri, Oliver Bracko, Robert Nahouraii

**Affiliations:** ^1^Department of Biology, University of Miami, Coral Gables, FL, United States; ^2^Department of Neurology, University of Miami-Miller School of Medicine, Miami, FL, United States; ^3^Mecklenburg Neurology Group, Charlotte, NC, United States; ^4^Mecklenburg Epilepsy and Sleep Center, Charlotte, NC, United States

**Keywords:** Epworth score, Epworth Sleepiness Scale, apnea-hypopnea index, inconsistencies, obstructive sleep apnea, excessive daytime sleepiness

## Abstract

**Introduction:**

A common practice in clinical settings is the use of the Epworth Sleepiness Scale (ESS) and apnea-hypopnea index (AHI) to demonstrate the severity of obstructive sleep apnea (OSA). However, several instances were noted where there were discrepancies in the reported severity between Epworth scores and AHI in our patient sample, prompting an investigation into whether OSA severity as demonstrated by AHI or predicted by ESS quantification of sleepiness is primarily responsible for inconsistencies.

**Methods:**

Discrepancies were examined between Epworth scores and AHI by categorizing patients into two categories of inconsistency: individuals with either ESS < 10 and AHI ≥ 15 events/h or ESS ≥ 10 and AHI < 15 events/h. The potential influence of sex on these categories was addressed by assessing whether a significant difference was present between mean Epworth scores and AHI values for men and women in the sample. We investigated BMI both by itself as its own respective variable and with respect to the sex of the individuals, along with a consideration into the role of anxiety. Furthermore, we tested anxiety with respect to sex.

**Results:**

In the first category of inconsistency the average ESS of 5.27 ± 0.33 suggests a normal level of daytime sleepiness. However, this contrasts with the average AHI of 32.26 ± 1.82 events/h which is indicative of severe OSA. In the second category the average ESS of 14.29 ± 0.47 suggests severe daytime sleepiness, contradicting the average AHI of 9.16 ± 0.44 events/h which only indicates mild OSA. Sex, BMI (both as a variable by itself and with respect to sex), and anxiety (both as a variable by itself and with respect to sex) contributed to observed inconsistencies.

**Conclusion:**

The findings of our study substantiate our hypothesis that Epworth scores should be de-emphasized in the assessment of OSA and a greater importance should be placed on measures like AHI. While Epworth scores offer insights into patients’ daytime sleepiness levels and the perceived severity of their OSA, the inconsistencies highlighted in our results when compared to AHI-based OSA severity underscore their potential inaccuracy. Caution is advised when utilizing Epworth scores for evaluating OSA severity in clinical settings.

## Introduction

Obstructive sleep apnea (OSA) is a common condition characterized by a complete (apnea) or partial collapse (hypopnea) of the upper respiratory tract (1). The lack of sleep one experiences due to OSA leads to fatigue throughout the day, or excessive daytime sleepiness (EDS) (2). Epworth scores are a measurement of the severity of EDS and are determined by the Epworth Sleepiness Scale (ESS). The scale ranges from 0 to 24 with higher scores (≥10) representing severe EDS that may require an opinion by a sleep specialist (3, 4). Its current use in clinical settings can be attributed to the questionnaire being inexpensive and quick to administer (3, 5). In addition, a report regarding its validity shows a significant positive correlation between Epworth scores and the respiratory disturbance index (RDI), which was calculated as the total number of apneas and hypopneas leading to a >3% drop regarding oxygen saturation per hour of sleep (3). According to the American Academy of Sleep Medicine Scoring Manual, the RDI is defined as the sum of the total number of apneas, hypopneas and respiratory effort-related arousals (RERAs) per hour of sleep (6, 7). RERAs are reductions in airflow that do not meet the criteria for apneas or hypopneas and are characterized by increased respiratory effort lasting ≥10 s (7).

The apnea-hypopnea index (AHI) represents the combined average of apneas and hypopneas that occur per hour of sleep. A mild AHI ranges from 5 to 15 apnea/hypopnea events per hour, a moderate AHI ranges from 15 to 30 events per hour, and a severe AHI ranges from >30 events per hour (8). This measurement is determined by polysomnography where the patient undergoes a sleep study to record parameters including brain waves, heart rate, breathing, movement in the eyes and legs, and blood oxygen levels (9). The AHI has been regarded as the gold standard for determining OSA (10). Generally, the severity of OSA is evaluated based on AHI, usually following AASM guidelines (11). Epworth score is part of the clinical evaluation of patients who potentially have OSA and is primarily used to measure the level of patients’ daytime sleepiness (3) and has also been assessed regarding its ability to predict OSA severity (5).

In the Mecklenburg Neurology patient sample, numerous cases were observed where severe AHI or Epworth score was reported, but the accompanying Epworth score or AHI did not agree in terms of severity. This inconsistency between ESS and AHI has been shown in previous studies (12–15). Ma et al. compared the severity of several variables and reports no difference between the mean AHI of an EDS vs. non-EDS group (12). A similar study conducted by Gabryelska et al. compared REM-dependent vs. REM-independent groups and observed similar ESS despite the REM-dependent group having two times lower median AHI (13). Sharkey et al. examined the use of Epworth scores in the assessment of patients’ OSA who were considering bariatric surgery to treat their obesity and determined that subjective measures did not differ by AHI group (14). Individuals with and without OSA (as determined by AHI ≥ 5 events/h) reported ESS scores that had no significant difference in a study conducted by Sunwoo et al. (15). We further analyzed the data in our patient sample to better understand and determine which variable is primarily responsible for these observed inconsistencies.

## Materials and methods

This was a retrospective chart review of a clinical sample consisting of 200 patients (*n* = 200) that had an AHI ≥ 5 events/h which is diagnosable for OSA. The exact length of OSA is unclear since none of these patients were previously diagnosed with OSA prior to receiving a sleep study at the study site. All information was obtained from clinical observations and electronic medical records of patients who came to the study site(s) for treatment of their EDS and fatigue and received CPAP, BiPAP machine or oral appliances based on the severity of OSA and patients’ tolerability to certain treatments. The study included patient medical information ranging from February 2022 to June 2023.

Two categories of inconsistency were considered to see the proportion of our patient sample that had an Epworth score or AHI that did not agree with the other variable in terms of severity: category 1 consisted of patients with ESS < 10 and AHI ≥ 15 events/h and category 2 had patients with ESS ≥ 10 and AHI < 15 events/h. Category 1 is classified by AHI ≥ 15 specifically when ESS < 10 because ESS < 10 is classified as experiencing little or average amount of daytime sleepiness, and having an Epworth score indicating anything less than an excessive amount of daytime sleepiness would be inconsistent for a patient who also has at least moderate OSA (AHI ≥ 15). Category 2 is classified by ESS ≥ 10 specifically for when AHI < 15 events/h because AHI < 15 events/h is classified as having mild OSA, and having an AHI indicating anything less than moderate to severe OSA would be inconsistent for a patient who is also experiencing an excessive amount of daytime sleepiness. The potential impact of sex on the inconsistencies observed between Epworth scores and AHI was evaluated by comparing the average Epworth scores and AHI between men (*n* = 93) and women (*n* = 107) in the cohort. A significant difference (*p* < 0.05) would indicate a notable disparity. If a significant difference is found in the entire sample, not just among those with inconsistencies, the next step is to examine the distribution of men and women within the inconsistency categories. This could help to determine the extent to which sex influences the severity of these inconsistencies. In addition, BMI was investigated in this context where ≥30.0 kg/m^2^ was classified as obese to determine whether BMI serves as a contributing factor both by itself as its own respective variable and with respect to sex to observed inconsistencies (*n* = 146 for obese; *n* = 54 for non-obese). Anxiety—which was already diagnosed by each patients’ primary care physician or referring physician—was also considered as psychological factors can make individuals perceive increased levels of fatigue throughout the day thus influencing Epworth scores (*n* = 86 for individuals with anxiety; *n* = 114 for individuals without anxiety). Anxiety with respect to sex and BMI was examined as well as it relates to uncovering contributors to observed inconsistencies.

Epworth scores were determined through evaluation of each patients’ administered ESS questionnaire. Each patient indicated their chance of falling asleep in different situations (sitting and reading; watching TV; sitting inactive in a public place; as a passenger in a car for an hour without a break; lying down to rest in the afternoon when circumstances permit; sitting and talking to someone; sitting quietly after a lunch without alcohol; in a car, while stopped for a few minutes in traffic) on a scale of 0–3 with 0 indicating no chance of dozing, 1 indicating slight chance of dozing, 2 indicating moderate chance of dozing, and 3 indicating high chance of dozing. AHI for each patient was determined through a full polysomnography performed in a sleep center monitored by a registered sleep technician where the total number of apneas and hypopneas were observed during the nighttime sleep. Each patient’s recording lasted at least 6 h. The sleep study was reviewed by a board-certified sleep physician the following day. A full night polysomnogram recorded the standard physiological parameters including electroencephalogram (EEG), electrooculogram (EOG), electromyography (EMG), electrocardiography (EKG), and nasal and oral airflow. Respiratory parameters of chest and abdominal movements were recorded with Piezo-Crystal motion transducers. Oxygen saturation was recorded by pulse oximetry. The sleep technician recorded the total duration of the sleep study and total sleep time, position that the patient slept, sleep stages, sleep latency, sleep efficiency and wakefulness. Patients with AHI of at least 5 events/h–minimum AHI required for sleep apnea diagnosis (8)–were included in the study. Patients were excluded from the study if they were below the age of 18, exhibited severe back pain causing an inability to sleep on their back, or were unable to complete the sleep study (i.e., sleep study failed to exceed 2 h).

One-way analysis of variance (ANOVA) followed by a *Post Hoc* Tukey Honestly Significant Difference (HSD) test was performed to determine which pairs of means for Epworth score and AHI, respectively, present a significant difference when comparing means across obese and non-obese individuals with respect to sex in the entire sample (not limited to individuals only presenting inconsistencies between Epworth score and AHI) as well as individuals with and without anxiety with respect to sex. Comparisons between sex, BMI, and anxiety were performed with Student’s *t*-test to determine if statistical difference was *p* < 0.05.

This study is exempt from IRB review pursuant to the terms of the U.S. Department of Health and Human Services’ Policy for Protection of Human Research Subjects at 45 C.F.R. §46.104(d). Sterling IRB has determined that Category 4 Exemption (DHHS) applies to this study.

## Results

Exactly half of the patients in our sample (*n* = 100) reported EDS (represented by their reported Epworth score) inconsistent with the severity of their OSA as determined by a polysomnography (represented by AHI). 29.5% of patients (*n* = 59) reported that they experience little or average amounts of daytime sleepiness (ESS < 10) while their AHI revealed they have moderate to severe OSA (AHI ≥ 15 events/h). 20.5% of patients (*n* = 41) reported EDS suggesting they experience excessive daytime sleepiness (ESS ≥ 10) while their AHI revealed they only have mild OSA (AHI < 15 events/h) (Table 1).

**Table 1:** Patients with conflicting Epworth scores and AHI.

	ESS < 10 AHI ≥ 15 events/h	ESS ≥ 10 AHI < 15 events/h
# of Patients (%)	*n* = 59 (29.5%)	*n* = 41 (20.5%)
Average Epworth score	5.27 ± 0.33	14.29 ± 0.47
Average AHI (events/h)	32.26 ± 1.82	9.16 ± 0.44

Sex was investigated to see if it contributes to the inconsistencies shown in Table 1. The mean Epworth score for men (in the entire sample, not only those reporting conflicting Epworth scores and AHI) was 8.47 ± 0.50 and 9.05 ± 0.53 for women (*p* > 0.05; *p* = 0.4400). The difference between Epworth scores was not significant, but when comparing AHI the average for men was 29.64 ± 2.7 events/h and 19.00 ± 1.75 events/h for women (*p* < 0.05; *p* = 0.0008), indicating statistical significance. The distribution of men and women in category 1 where ESS < 10 and AHI ≥ 15 events/h was 28 men (47.5%) and 31 women (52.5%). For category 2 where ESS ≥ 10 and AHI < 15 events/h, there were 12 men (29.3%) and 29 women (70.7%) (Table 2).

**Table 2:** Comparison of mean Epworth scores and AHI between men and women.

	Men	Women	*p*-value (*p* < 0.05, significant)
Average Epworth score	8.47 ± 0.50	9.05 ± 0.53	*p* = 0.4400
Average AHI (events/h)	29.64 ± 2.70	19.00 ± 1.75	*p* = 0.0008
Prevalence in ESS < 10 and AHI ≥ 15 events/h (%)	*n* = 28 (47.5%)	*n* = 31 (52.5%)	–
Prevalence in ESS ≥ 10 and AHI < 15 events/h (%)	*n* = 12 (29.3%)	*n* = 29 (70.7%)	–

BMI was considered as contributing to the inconsistencies shown in Table 1. The mean Epworth score for obese individuals (i.e., BMI ≥ 30 kg/m^2^) was 9.03 ± 0.42 and 8.09 ± 0.77 for non-obese individuals (*p* > 0.05; *p* = 0.2588). The difference between Epworth scores was not significant, but when comparing AHI the average for obese individuals was 26.79 ± 2.08 events/h and 16.25 ± 1.45 events/h for non-obese individuals (*p* < 0.05; *p* = 0.0034), indicating statistical significance. The distribution of obese and non-obese individuals in category 1 where ESS < 10 and AHI ≥ 15 events/h was 44 obese (74.6%) and 15 non-obese (25.4%). For category 2 where ESS ≥ 10 and AHI < 15 events/h, there were 25 obese (61.0%) and 16 non-obese (39.0%) individuals (Table 3).

**Table 3:** Comparison of mean Epworth scores and AHI between obese and non-obese individuals.

	BMI ≥ 30 kg/m^2^ (i.e., obese)	BMI < 30 kg/m^2^	*p*-value (*p* < 0.05, significant)
Average Epworth score	9.03 ± 0.42	8.09 ± 0.77	*p* = 0.2588
Average AHI (events/h)	26.79 ± 2.08	16.25 ± 1.45	*p* = 0.0034
Prevalence in ESS < 10 and AHI ≥ 15 events/h (%)	*n* = 44 (74.6%)	*n* = 15 (25.4%)	–
Prevalence in ESS ≥ 10 and AHI < 15 events/h (%)	*n* = 25 (61.0%)	*n* = 16 (39.0%)	–

Mean AHI was determined for obese and non-obese individuals with respect to sex and then compared through *Post Hoc* Tukey HSD following an initial one-way ANOVA test (*F* = 9.5630 which corresponds to *p* < 0.00001). 35.75 events/h was determined as the mean AHI for obese men, 17.44 events/h for non-obese men, 20.19 events/h for obese women, and 14.67 events/h for non-obese women. The *Post Hoc* Tukey HSD then produced *Q* = 5.32 (*p* = 0.0013) when comparing the mean AHI between obese men and non-obese men, *Q* = 4.52 (*p* = 0.0088) between obese men and obese women, *Q* = 6.12 (*p* = 0.0001) between obese men and non-obese women, *Q* = 0.80 (*p* = 0.9426) between non-obese men and obese women, *Q* = 0.81 (*p* = 0.9411) between non-obese men and non-obese women, and *Q* = 1.60 (*p* = 0.6695) between obese women and non-obese women (Table 4).

**Table 4:** Pairwise comparisons from *Post Hoc* Tukey HSD (AHI) for obese and non-obese individuals with respect to sex.

Pairwise comparisons		HSD_0.05_ = 12.6233 HSD_0.01_ = 15.3640	*Q*_0.05_ = 3.6645 *Q*_0.01_ = 4.4601
*G*_1_:*G*_2_	*M*_1_ = 35.75 *M*_2_ = 17.44	18.31	*Q* = 5.32 (*p* = 0.0013)
*G*_1_:*G*_3_	*M*_1_ = 35.75 *M*_3_ = 20.19	15.56	*Q* = 4.52 (*p* = 0.0088)
*G*_1_:*G*_4_	*M*_1_ = 35.75 *M*_4_ = 14.67	21.08	*Q* = 6.12 (*p* = 0.0001)
*G*_2_:*G*_3_	*M*_2_ = 17.44 *M*_3_ = 20.19	2.75	*Q* = 0.80 (*p* = 0.9426)
*G*_2_:*G*_4_	*M*_2_ = 17.44 *M*_4_ = 14.67	2.77	*Q* = 0.81 (*p* = 0.9411)
*G*_3_:*G*_4_	*M*_3_ = 20.19 *M*_4_ = 14.67	5.52	*Q* = 1.60 (*p* = 0.6695)

The values for HSD_0.5_, HSD_0.1_, *Q*_0.5_, and *Q*_0.1_ indicate the values that must be met or exceeded for HSD and *Q*, respectively, to observe a significant difference between the means of the two groups being compared. The values for HSD_0.5_ and *Q*_0.5_ are the values that must be met for a significance level of *p* < 0.05. The values for HSD_0.1_ and *Q*_0.1_ are the values that must be met for a significance level of *p* < 0.01. The values shown in the HSD column are the difference between the means of the two groups being compared. Obese men are classified as *G*_1_ (“group 1”) and the mean AHI for this group is referred to as *M*_1_ (“mean 1”). Non-obese men are *G*_2_ and *M*_2_. Obese women are *G*_3_ and *M*_3_. Non-obese women are *G*_4_ and *M*_4_.

The proportion of men and women that were obese and non-obese in both categories was considered to see if BMI with respect to sex may contribute to observed inconsistencies between Epworth scores and AHI. The proportion of obese men in category 1 where ESS < 10 and AHI ≥ 15 events/h was 19 (67.9%) and for non-obese was 9 (32.1%), and for women the proportion was 25 obese (80.6%) and 6 non-obese (19.4%). The proportion of obese men in category 2 where ESS ≥ 10 and AHI < 15 events/h was 6 (50.0%) and for non-obese was 6 (50.0%), and regarding women in this category the proportion was 19 obese (65.5%) and 10 non-obese (34.5%) (Table 5).

**Table 5:** Proportion of obese and non-obese individuals with respect to sex within categories of inconsistency.

	BMI ≥ 30 kg/m^2^ (i.e., obese)	BMI < 30 kg/m^2^	*p*-value (*p* < 0.05, significant)
# of Men in ESS < 10 and AHI ≥ 15 events/h (%)	*n* = 19 (67.9%)	*n* = 9 (32.1%)	–
# of Women in ESS < 10 and AHI ≥ 15 events/h (%)	*n* = 25 (80.6%)	*n* = 6 (19.4%)	–
# of Men in ESS ≥ 10 and AHI < 15 events/h (%)	*n* = 6 (50.0%)	*n* = 6 (50.0%)	–
# of Women in ESS ≥ 10 and AHI < 15 events/h (%)	*n* = 19 (65.5%)	*n* = 10 (34.5%)	–

Anxiety was considered to see if psychological factors contribute to observed inconsistencies shown in Table 1 by Epworth scores being increased. The mean Epworth score for individuals reporting anxiety was 9.64 ± 0.57 and 8.13 ± 0.48 for those without (*p* < 0.05; *p* = 0.0430). The difference between Epworth scores was significant here, but when comparing AHI the average for those with anxiety was 23.61 ± 2.11 events/h and 24.20 ± 2.33 events/h for those without (*p* > 0.05; *p* = 0.8562), failing to indicate statistical significance. The distribution of category 1 where ESS < 10 and AHI ≥ 15 events/h was 25 with anxiety (42.4%) and 34 without anxiety (57.6%). For category 2 where ESS ≥ 10 and AHI < 15 events/h, there were 20 with anxiety (48.8%) and 21 without anxiety (51.2%) (Table 6).

**Table 6:** Comparison of mean Epworth scores and AHI between individuals with anxiety and those without.

	Anxiety	No anxiety	*p*-value (*p* < 0.05, significant)
Average Epworth score	9.64 ± 0.57	8.13 ± 0.48	*p* = 0.0430
Average AHI (events/h)	23.61 ± 2.11	24.20 ± 2.33	*p* = 0.8562
Prevalence in ESS < 10 and AHI ≥ 15 events/h (%)	*n* = 25 (42.4%)	*n* = 34 (57.6%)	–
Prevalence in ESS ≥ 10 and AHI < 15 events/h (%)	*n* = 20 (48.8%)	*n* = 21 (51.2%)	–

Mean AHI was determined for individuals with and without anxiety with respect to sex and then compared through *Post Hoc* Tukey HSD following an initial one-way ANOVA test (*F* = 4.4768 which corresponds to *p* = 0.00458). 26.68 events/h was determined as the mean AHI for men with anxiety, 31.79 events/h for men without anxiety, 21.07 events/h for women with anxiety, and 17.38 events/h for women without anxiety. The *Post Hoc* Tukey HSD then produced *Q* = 1.61 (*p* = 0.6666) when comparing the mean AHI between men with anxiety and men without anxiety, *Q* = 1.77 (*p* = 0.5953) between men with anxiety and women with anxiety, *Q* = 2.93 (*p* = 0.1656) between men with anxiety and women without anxiety, *Q* = 3.38 (*p* = 0.0826) between men without anxiety and women with anxiety, *Q* = 4.54 (*p* = 0.0083) between men without anxiety and women without anxiety, and *Q* = 1.16 (*p* = 0.8439) between women with anxiety and women without anxiety (Table 7).

**Table 7:** Pairwise comparisons from *Post Hoc* Tukey HSD (AHI) for individuals with and without anxiety with respect to sex.

Pairwise comparisons		HSD_0.05_ = 11.6302 HSD_0.01_ = 14.1552	*Q*_0.05_ = 3.6645 *Q*_0.01_ = 4.4601
*G*_1_:*G*_2_	*M*_1_ = 26.68 *M*_2_ = 31.79	5.11	*Q* = 1.61 (*p* = 0.6666)
*G*_1_:*G*_3_	*M*_1_ = 26.68 *M*_3_ = 21.07	5.61	*Q* = 1.77 (*p* = 0.5953)
*G*_1_:*G*_4_	*M*_1_ = 26.68 *M*_4_ = 17.38	9.30	*Q* = 2.93 (*p* = 0.1656)
*G*_2_:*G*_3_	*M*_2_ = 31.79 *M*_3_ = 21.07	10.72	*Q* = 3.38 (*p* = 0.0826)
*G*_2_:*G*_4_	*M*_2_ = 31.79 *M*_4_ = 17.38	14.41	*Q* = 4.54 (*p* = 0.0083)
*G*_3_:*G*_4_	*M*_3_ = 21.07 *M*_4_ = 17.38	3.69	*Q* = 1.16 (*p* = 0.8439)

The values for HSD_0.5_, HSD_0.1_, *Q*_0.5_, and *Q*_0.1_ indicate the values that must be met or exceeded for HSD and *Q*, respectively, to observe a significant difference between the means of the two groups being compared. The values for HSD_0.5_ and *Q*_0.5_ are the values that must be met for a significance level of *p* < 0.05. The values for HSD_0.1_ and *Q*_0.1_ are the values that must be met for a significance level of *p* < 0.01. The values shown in the HSD column are the difference between the means of the two groups being compared. Men with anxiety are classified as *G*_1_ (“group 1”) and the mean AHI for this group is referred to as *M*_1_ (“mean 1”). Men without anxiety are *G*_2_ and *M*_2_. Women with anxiety are *G*_3_ and *M*_3_. Women without anxiety are *G*_4_ and *M*_4_.

The proportion of men and women with and without anxiety in both categories was considered to see if anxiety with respect to sex may contribute to observed inconsistencies between Epworth scores and AHI. The proportion of men with anxiety in category 1 where ESS < 10 and AHI ≥ 15 events/h was 13 (46.4%) and for men without anxiety was 15 (53.6%), and for women the proportion was 12 with anxiety (38.7%) and 19 without anxiety (61.3%). The proportion of men with anxiety in category 2 where ESS ≥ 10 and AHI < 15 events/h was 6 (50.0%) and for men without anxiety was 6 (50.0%), and regarding women in this category the proportion was 14 with anxiety (48.3%) and 15 without anxiety (51.7%) (Table 8).

**Table 8:** Proportion of individuals with and without anxiety with respect to sex within categories of inconsistency.

	Anxiety	No anxiety	*p*-value (*p* < 0.05, significant)
# of Men in ESS < 10 and AHI ≥ 15 events/h (%)	*n* = 13 (46.4%)	*n* = 15 (53.6%)	–
# of Women in ESS < 10 and AHI ≥ 15 events/h (%)	*n* = 12 (38.7%)	*n* = 19 (61.3%)	–
# of Men in ESS ≥ 10 and AHI < 15 events/h (%)	*n* = 6 (50.0%)	*n* = 6 (50.0%)	–
# of Women in ESS ≥ 10 and AHI < 15 events/h (%)	*n* = 14 (48.3%)	*n* = 15 (51.7%)	–

## Discussion

Our retrospective chart review of the Mecklenburg Neurology patient sample reveals inconsistencies between Epworth scores and AHI in regards to their respective severities. In regards to the average ESS and AHI for the ESS < 10 and AHI ≥ 15 events/h category, the average ESS of 5.27 ± 0.33 indicates a normal amount of daytime sleepiness which is inconsistent with the average AHI of 32.26 ± 1.82 events/h which indicates severe OSA (Table 1). For the ESS ≥ 10 and AHI < 15 events/h category, the average ESS of 14.29 ± 0.47 is suggestive of severe daytime sleepiness, but the average AHI of 9.16 ± 0.44 events/h only indicates mild OSA and is thus inconsistent with the Epworth score variable (Table 1).

Sex may have influenced the average severity of the AHI variable in each category since a significant difference between average AHI for men (*n* = 93) and women (*n* = 107) was observed (Table 2). Since there were more men in the ESS < 10 and AHI ≥ 15 events/h category compared to the number of men in the ESS ≥ 10 and AHI < 15 events/h category, the average AHI may have been increased and thus contributed to a greater inconsistency when compared to the severity suggested by Epworth scores for that category (Table 2). Men are known to exhibit worse OSA when measured by AHI (16, 17), which was observed in our study. This may be due to men reportedly experiencing increased apneas and hypopneas during NREM sleep (18), which constitutes 75–80% of total sleep (19). Studies also report a higher prevalence of OSA in men (20, 21) with that prevalence becoming comparable when considering post-menopausal women (as well as those undergoing hormone replacement therapy post-menopause) who have a higher prevalence of OSA compared to premenopausal women (22, 23). Nevertheless, while a significant difference between mean AHI for men and women was observed, the mean Epworth scores were not statistically significant regarding their difference which presents an inconsistency between the two variables. These observed inconsistencies have been noted in other studies that report a weak correlation between Epworth scores and AHI (5, 24, 25). It is important to note, though, that there are studies reporting a significant positive correlation between ESS values and AHI (significant positive correlation observed for both ESS self-administered by the patient and ESS administered by the doctor) (26) so Epworth scores can still be expected to increase with AHI and serve as a predictor of OSA severity. Another study found that AHI had a significant correlation with the ESS questionnaire but not with the STOP-BANG questionnaire (27), which screens for OSA based on major risk factors including snoring, tiredness, observed apnea, blood pressure, BMI, age, neck size, gender (28).

BMI was another demographic factor considered potentially contributing to the inconsistencies observed. A significant difference was observed between mean AHI for obese (*n* = 146) vs. non-obese individuals (*n* = 54) as well as a higher proportion of obese individuals in the ESS < 10 and AHI ≥ 15 events/h category, which indicates that an increased amount of obese individuals in the first category may be contributing to the increased AHI observed in that category and may exacerbate the inconsistency between Epworth scores and AHI (Table 3). The significant difference observed in AHI here is expected as previous studies show how AHI increases with BMI (29–31). One reason for this correlation could be due to obese individuals experiencing twice as many sleep-related problems compared to non-obese individuals (29), and a potential reason for this report may be because of increased fat depositions (one study reports 42% more depositions compared to levels in normal patients) (32) in the neck of obese patients experiencing upper airway obstructions due to their OSA, which subsequently makes the airway more vulnerable to collapse and leads to apnea (33). In addition, increased levels of leptin are seen in both obese patients and those with OSA with the level of leptin serving as an indicator of OSA severity (29). This suggests that increased leptin may serve as a linking condition between obesity and OSA. Obesity and OSA severity have been shown to correlate in longitudinal studies as well (34, 35). A study conducted by Ip et al. reports a positive correlation between OSA severity (measured by AHI in this study) and BMI throughout a 5-year duration (34). A reduction in BMI due to intensive lifestyle interventions and Roux-en-Y gastric bypass has also been observed to reduce AHI thus reducing OSA severity (surgery was more effective for mitigating OSA) (35). Nearly identical mean Epworth scores were reported for obese and non-obese patients despite the strong difference observed for AHI; previous studies show that those with obesity achieve significantly less sleep (29), which should be represented by an increase in EDS supported by significantly higher Epworth scores observed on average when compared to the Epworth scores of non-obese individuals. This was not observed, though, rather we report a difference of only ~1 in regards to the difference between mean Epworth scores for obese and non-obese individuals. It is also worth noting that obesity can be associated with EDS even in subjects without OSA (36). Our data concerning BMI supports an increased accuracy of AHI compared to Epworth scores in our patient sample specifically in the context of obesity.

Pairwise comparisons from *Post Hoc* Tukey HSD for obese and non-obese individuals with respect to sex were conducted to compare mean Epworth scores and AHI determined by an initial one-way ANOVA test. A significant difference was not observed following one-way ANOVA when comparing mean Epworth scores between obese men, non-obese men, obese women and non-obese women and thus a *Post Hoc* Tukey HSD was not performed. When comparing these groups in regards to their mean AHI, though, statistically significant results were observed from both the initial one-way ANOVA test and when comparing the mean AHI of obese men to that of the rest of the groups (Table 4). Obese men reported a mean Epworth score of 9.21 and mean AHI of 35.75 events/h which falls directly into the ESS < 10 and AHI ≥ 15 events/h category of inconsistency. In addition, their mean AHI indicated the most severe case of OSA (i.e., highest AHI) and appeared to be significantly different from the rest of the groups, yet the Epworth score measurement does not represent this in that the mean Epworth score for obese men does not differ significantly from any of the other groups nor is it even the highest. Rather, non-obese women report the highest mean Epworth score of 9.57 despite them having the lowest mean AHI of 14.67 events/h. The observation of obese men associating with the first category of inconsistency and non-obese women having the highest mean Epworth score despite also having the lowest mean AHI ultimately suggests that Epworth scores are primarily responsible for the observed inconsistencies when examining BMI in relation to sex within our patient sample. The increased AHI observed in obese men relative to other groups is supported by a study from Liu et al. where they showed that men with increased BMI suffer from a more severe increase in AHI (32%) compared to women (26%) (37). Newman et al. also indicates that changes in BMI are associated with an increase or decrease regarding the severity of OSA (as measured by RDI) and is stronger in men (38). In regards to the interaction between weight change and sex, Newman et al. shows a significant interaction between the two (*p* = 0.002) which further supports different effects on RDI due to weight change in men and women, respectively (38). Several studies demonstrate a weak relationship between AHI and ESS specifically in obese patients despite a strong association between obesity and OSA (36, 39–42) similar to what we observed in our patient sample.

The sex of the individuals when looking at the proportion of obese and non-obese patients in each category of inconsistency was considered to determine if a high prevalence of obese or non-obese men or women is present in either category which could lead to an explanation for the observed inconsistencies. A higher proportion of obese men and women in each category was observed except for the number of men in the second category where obese and non-obese men were present in equal amounts (Table 5). In the ESS < 10 and AHI ≥ 15 events/h category it can reasonably be assumed that an increased proportion of obese men contributed to the increased AHI in this category of inconsistency since Table 4 shows obese men as having the highest AHI of 35.75 events/h. This finding aligns with our earlier discussed outcomes shown in Table 2 where men had a notable elevation in AHI compared to women. In addition, the data in Table 3 showed that obese individuals have significantly higher AHI than non-obese individuals. Thus, when looking at obese men we expect increased AHI which would contribute to the high AHI seen in the first category of inconsistency. It is also important to note that there were much more obese women in this category compared to the amount of non-obese women (61.2% more obese women) which could have contributed to increased AHI since obese women have the next highest AHI of 20.19 events/h and thus ultimately contributed to a more severe inconsistency. The increased proportion of obese men having a greater contribution to the elevated AHI is supported by a previous study conducted by Huang et al. where they observed higher severity of OSA in obese male patients compared to obese female patients (i.e., as BMI increased, AHI significantly increased more in the male than female patients) and ultimately reached the conclusion that both increased body weight and obesity play a more significant role in contributing to OSA in male patients (43). In the ESS ≥ 10 and AHI < 15 events/h category a higher proportion of non-obese women would be expected since they reported the highest mean Epworth score of 9.57, but instead there was a higher proportion of obese women who reported a mean Epworth score of 8.90. Thus, a reasonable assumption that the observed proportion of obese and non-obese individuals with respect to sex in this category of inconsistency significantly contributed to the ESS ≥ 10 cannot be derived.

Psychological factors have been shown to influence the perception of daytime sleepiness thus influencing Epworth scores even if other measurements of OSA support less severe sleep apnea (44, 45). The presence of anxiety was considered in a number of patients and a significant difference in mean Epworth score between individuals with (*n* = 86) and without anxiety (*n* = 114) was observed where those with anxiety reported higher Epworth scores (Table 6). Our sample showing increased Epworth scores for individuals with anxiety supports the claim that people with psychological disorders may misinterpret their level of daytime sleepiness and thus report influenced Epworth scores. In regards to the prevalence of anxious vs. non-anxious individuals in each category of inconsistency, the mean Epworth score and AHI in the ESS < 10 and AHI ≥ 15 events/h category may not have been influenced as much since there were less anxious individuals in the group compared to the number of non-anxious individuals (15.2% less anxious individuals) which is expected since this category deals with ESS < 10. In the ESS ≥ 10 and AHI < 15 events/h category, a higher proportion of non-anxious individuals was observed but only by 2.4%, so the increased Epworth score could be attributed to almost half of the individuals in this category having anxiety. A previous study conducted by González-Heredia et al. shows a majority of patients in an investigated sample that were subject to EDS having anxiety (44). Choueiry et al. conducted a similar study where the association between insomnia and anxiety was evaluated through the use of different questionnaires that reveal the severity of a patient’s daytime sleepiness, including the ESS, and found that 50.8% of the patient sample with clinically significant anxiety had EDS (46). An additional study supports a correlation between Epworth scores and anxiety severity (45), and another supports the influence of ESS scores by psychological factors including anxiety while measures like the multiple sleep latency test (MSLT) were not influenced by such (47), further supporting the idea that Epworth scores can be influenced due to anxiety. It is important to note that anxiety disorders such as generalized anxiety disorder and posttraumatic stress disorder are usually associated with insomnia (48), and increased EDS may be a subsequent result of the insomnia in anxious people (49).

Nguyen et al. conducted a study evaluating the variability of Epworth scores using a test–retest method to assess their potential impact on clinical decision-making. Patients completed the questionnaire twice, 71 days apart, to determine if they consistently received the same score. Results showed significant variability, with 41% of patients exhibiting a difference of 3 between their first and second ESS tests, indicating poor test–retest reliability and high variability of ESS scores within clinical populations (50). This is in contrast to Murray Johns’ (individual who originally proposed the ESS assessment) observations during his own study of ESS reliability where he observed only a 0.2 average difference between the 2 scores when the questionnaire was administered 5 months apart, indicating good test–retest reliability (51). In addition, Nguyen et al. constructed a Bland–Altman plot showing the mean ESS scores against the difference in the two scores and it suggested low test–retest reliability of ESS specifically in a population being investigated for OSA. A similar study was conducted by Taylor et al. but, rather than 2, they tracked 3 different Epworth scores per person from general practitioner ESS, overnight oximetry ESS, and sleep specialist ESS and they observed a poor test–retest as well (52), further supporting the conclusions drawn by Nguyen et al. Another study observed low test–retest reliability even when the ESS test is repeatedly administered on the same day (53).

Anxiety was considered with respect to sex and BMI by way of multiple comparisons through *Post Hoc* Tukey HSD following one-way ANOVA. The initial one-way ANOVA tests dedicated to comparing mean Epworth scores were insignificant and thus *Post Hoc* Tukey HSD was not performed. On the other hand, the one-way ANOVA for anxiety with respect to sex when comparing mean AHI did report significance which can be attributed to men without anxiety having a significantly different AHI compared to that of women without anxiety (Table 7). This reinforces our previously reported results in that anxiety does not significantly contribute to AHI (Table 6), rather sex is the primary factor; more specifically, men in our patient sample have significantly increased AHI relative to women (Table 2). Regarding inconsistencies between ESS and AHI, men without anxiety report the highest AHI that specifically differs significantly from women without anxiety, yet their Epworth score does not and is the lowest among the groups (similar to what was observed with obese men in our patient sample). Men and women with anxiety report the highest mean Epworth scores of 9.13 and 10.06, respectively, which could be attributed to the significant difference between Epworth scores in patients with and without anxiety in our patient sample (Table 6) meaning that the presence of anxiety could have influenced Epworth scores in these groups. The observation of men without anxiety having both the highest AHI and the lowest Epworth scores, while individuals with anxiety report higher mean Epworth scores, suggests that ESS may be influenced by anxiety and serve as the primary contributor to inconsistencies in our patient sample. The absence of an influence by anxiety on AHI has been shown in a study conducted by Lee et al. where OSA severity had no relation to patients’ history of psychiatric diagnoses (i.e., depression and anxiety) (54). Another study shows that OSA severity (as measured by either AHI or RDI) is not related to comorbid anxiety (55). Both studies support a significant association between ESS and anxiety with anxiety measured by the Generalized Anxiety Disorder-7 (54) and State–Trait Anxiety Inventory-State Scale (55). As it relates to the proportion of individuals with and without anxiety with respect to sex (Table 8), each category of inconsistency contains more individuals without anxiety (with the exception of men in category 2 where ESS ≥ 10 and AHI < 15 events/h). This observation is supported by Bjorvatn et al. where they report a fewer number of individuals with anxiety as OSA severity increased (56), and in our patient sample we see the largest difference between the prevalence of individuals with and without anxiety (38.7% with anxiety vs. 61.3% without anxiety) in the category of inconsistency where AHI ≥ 15 (i.e., increased OSA severity). Interestingly, while the one-way ANOVA for anxiety with respect to BMI when comparing mean AHI produced a significant overall *F*-value and associated *p*-value (*F* = 2.9233 and *p* = 0.03509), the *Post Hoc* Tukey HSD did not reveal any significant pairwise differences.

Limitations of this study include that the data was collected from a single practice which may impact its generalization. In addition, certain factors that could have contributed to the observed results may not have been accounted for since this was a retrospective study on a clinical population (i.e., the study used information in patient charts that was not collected for the purpose of research). We recognize that this is a retrospective review, but the results could be important for future research.

## Conclusion

Our results in conjunction with those from the primary literature support our hypothesis that less emphasis should be put on Epworth scores when evaluating OSA and more focus on measures such as AHI. While Epworth scores appear to give insight into patients’ levels of daytime sleepiness and the severity of their OSA, the inconsistencies reported here when compared to OSA severity as determined by AHI reveal that these scores are more inaccurate. Demographic factors such as sex and BMI contribute to OSA severity, but Epworth scores are less accurate in representing this compared to AHI. In addition, psychological factors such as anxiety have been shown to influence and skew Epworth scores, which was observed in our patient sample. Our results suggest that Epworth scores should be used with caution when evaluating OSA severity in clinical settings.

Regarding future research, viewing variables in conjunction with each other rather than as variables by themselves could provide valuable insights into OSA severity of specific demographic groups that are clinically relevant. In addition, a new questionnaire should be developed that factors in an increased number of variables that could potentially be contributing to patients’ OSA including anxiety, BMI, etc., and an in-depth analysis of each variable should be achieved by this proposed questionnaire rather than solely utilizing yes/no questions or number scales so that we can provide patients with the most accurate diagnoses.

## Data availability statement

The raw data supporting the conclusions of this article will be made available by the authors, without undue reservation.

## Ethics statement

Ethical approval was not required for the study involving humans in accordance with the local legislation and institutional requirements. Written informed consent to participate in this study was not required from the participants or the participants’ legal guardians/next of kin in accordance with the national legislation and the institutional requirements.

## Author contributions

DA: Conceptualization, Data curation, Formal analysis, Methodology, Writing – original draft, Writing – review & editing. OB: Conceptualization, Validation, Writing – review & editing. RN: Conceptualization, Data curation, Project administration, Supervision, Validation, Writing – review & editing.
